# 

**Published:** 1879-08

**Authors:** 


*CONTENTS.*

| | PAGE |
|---|---|
| It Should Be Known | 1 |
| Poisons | 2 |
| Bites and Stings | 2 |
| Bugs and Fleas | 2 |
| Poison Plants | 2 |
| Mad Dogs | 2 |
| Bleeding | 3 |
| Fractures | 3 |
| Blows on the Head | 3 |
| Drowning | 3 |
| To Restore Breathing | 3 |
| To Imitate Breathing | 4 |
| General Observations | 4 |
| Caution | 4 |
| To Save from Drowning | 4 |
| Imaginary Diseases | 5 |
| Consumption | 6 |
| An Old Physician's Advice | 7 |
| Diseases of Children | 7 |
| Summer Complaints of Children—Symptoms and Treatment | 8 |
| Fever-and-Ague | 9 |
| Constipation | 9 |
| Care of Children | 10 |
| The Instinct of Nursing | 10 |
| Constipation of Children | 10 |
| Is Alum Poisonous? | 11 |
| The Secret of Success | 11 |
| Moles, Warts, and Birth-Marks | 12 |
| Duryees' Blow-Pipe Ore Furnace | 13 |
| A Valuable Bill of Fare | 15 |
| Bill of Fare for Diabetics | 16 |
| What the *World* said | 18 |
| Fefferale Flour | 18 |
| Health Foods in Philadelphia | 18 |
| The Health Food Co | 19 |
| Diabetes | 22 |
| The Universal Food | 24 |
| Cereal Coffee | 25 |
| Cold Blast (Attrition) Flour | 26 |
| Gluten | 29 |
| Fruit Food | 30 |
| Indigestion Relieved by Food | 30 |
| Opinions Differ | 30 |
| Health Foods in Hotels | 31 |

